# Sex Moderates the Effect of Aerobic Exercise on Some Aspects of Cognition in Cognitively Intact Younger and Middle-Age Adults

**DOI:** 10.3390/jcm8060886

**Published:** 2019-06-21

**Authors:** Yaakov Stern, Seonjoo Lee, David Predovan, Richard P. Sloan

**Affiliations:** 1 Department of Neurology and Taub Institute, Cognitive Neuroscience Division, Columbia University, New York, NY 10032, USA; dp2931@cumc.columbia.edu; 2 Mental Health Data Science, New York State Psychiatric Institute, New York, NY 10032, USA; sl3670@cumc.columbia.edu; 3 Department of Biostatistics, Columbia University, New York, NY 10032, USA; 4 Department of Psychiatry, Division of Behavioral Medicine, Columbia University, New York, NY 10032, USA; rps7@cumc.columbia.edu

**Keywords:** executive function, processing speed, BMI

## Abstract

We recently reported the results of a randomized, parallel-group, observer-masked, community-based clinical trial of 132 cognitively normal individuals aged 20–67 with below median aerobic capacity who were randomly assigned to one of two 6-month, four-times-weekly conditions: aerobic exercise and stretching/toning. We now assessed potential sex moderation on exercise-related changes in aerobic capacity, BMI and cognitive function. There was no sex moderation of the effect of aerobic exercise on aerobic capacity or BMI. We had previously reported an effect of aerobic exercise on executive function that was moderated by age. We found additional moderation by sex, such that in any age range men improved more than women. Processing speed showed significant sex moderation but not significant age moderation. In men, processing speed significantly improved by week 12 (b = 0.35, *p* = 0.0051), but the effect was diminished relative to week 12 at week 24 (b = 0.24, *p* = 0.0578). In women, there was no exercise effect at either time point (week 12: b = −0.06, *p* = 0.4156; week 24: b = −0.11, *p* = 0.1841). Men benefited cognitively more than women from aerobic exercise. This study highlights the importance of using sex-disaggregated analyses when assessing the impact of physical exercise intervention, and the need to ascertain the underlying mechanisms for differential cognitive benefit by sex.

## 1. Introduction

Animal and human studies indicate cognitive benefits from aerobic exercise across the lifespan, but controlled exercise studies in humans generally have been restricted to elderly individuals. Several meta-analyses in cognitively intact adults above age 55 have found aerobic exercise effects in the areas of attention, processing speed, executive function (EF), memory and working memory [1,2,3], with some suggesting strongest effects for EF [3], but there have also been negative conclusions [4,5]. To assess the impact of aerobic exercise on cognition in cognitively intact adults in a younger age range, we recently conducted and reported the results of a randomized controlled trial of aerobic exercise in sedentary adults age 20 through 67 [6]. We found that aerobic exercise was associated with improved aerobic capacity, decreased BMI, and an improvement in EF that interacted with age, such that the relative improvement in EF was greater with each year of age. 

The planned analyses for our trial did not incorporate sex moderation. Few randomized controlled trials have addressed the potential moderation of exercise effects by sex despite the fact that several observational and intervention studies have found sex differences in the cognitive benefit from physical exercise or physical activity [7,8,9,10,11,12,13]. Two meta-analyses [3,14] suggested that women might benefit from aerobic exercise more than men, but these analyses were not based on direct comparison of exercise benefit by sex. Rather, they simply classified studies based on their overall predominance of men or women [7,8,9,10,11,12,13]. Here we report additional analyses of the data from our study, evaluating the potential moderation of sex on the effect of aerobic exercise. 

We previously found that improvement in aerobic capacity mediated the effect of aerobic exercise on EF. To the extent that sex moderated the effects of aerobic exercise on cognition, it would be important to know whether this mediation was present and differed as a function of sex. We therefore planned to test these mediation effects if significant sex moderation was noted.

Understanding potential sex differences in cognitive benefit from aerobic exercise would have practical importance for guiding health behaviors. We hypothesized that there may be sex differences in the effect of aerobic exercise on cognition, but were unsure of the direction of this moderation. We also suspected that if there were significant sex moderation we might detect exercise benefits in cognitive domains other than EF that could not be detected when sex was not considered in the analysis. Finally, based on our previous results we predicted that improvement in cognitive function would be mediated by improved aerobic capacity, and that this mediation would be moderated by sex.

## 2. Experimental Section

### 2.1. Materials and Methods

Study methods were described in detail in our original report [6]. In this randomized, parallel-group, observer-masked, community-based clinical trial, 132 cognitively normal individuals aged 20–67 with below median aerobic capacity were randomly assigned to one of two 6-month, four-times-weekly conditions: aerobic exercise or stretching/toning. The study protocol and informed consent form were approved by the Institutional Review Board of New York State Psychiatric Institute. All participants provided written informed consent before enrollment. The trial was overseen by an independent data and safety monitoring board under the auspices of the National Institute on Aging. The trial was registered on Clinicaltrials.gov (Identifier: NCT01179958).

Study participants were healthy, cognitively intact, non-smoking, right-handed, sedentary, and habitual non-exercisers. A telephone screening determined that participants met basic inclusion/exclusion criteria. The Baecke Physical Activity Sports Score [15] excluded regular exercisers, defined as a score of >2 on this 5-point scale, from further participation. Potential participants then attended an in-person visit where they signed an informed consent document, and completed the Mattis Dementia Rating Scale [16]; participants with scores below 135 were excluded in order to ensure that participants were cognitively intact. They then underwent baseline cardiorespiratory exercise testing (CPET) to determine aerobic capacity (VO_2_ max). A normal baseline ECG was required. Any ischemic changes, abnormal blood pressure responses, or significant ectopy resulted in exclusion. 

CPET was used to ensure that participants had below median average fitness by American College of Sports Medicine standards (VO_2_ max <43, <38, <35, and <31 mL/kg/min for men age <40, 40–49, 50–59, and >59, respectively; <36, <34, <32, <25, and <24 mL/kg/min for women age <40, 40–49, 50–59, and >59 respectively). The baseline aerobic capacity testing was used to establish safe exercise parameters and heart rate (HR) targets for training.

Qualified participants then proceeded to the two-week run-in period where they attended their choice of YMCA fitness centers in Manhattan, three times/week for two weeks, performing a schedule of activities that did not include aerobic exercise. Only those who attended at least five sessions were then randomized into one of two conditioning programs: stretching/toning or aerobic exercise. A comprehensive report of the exercise programs using the Consensus on Exercise Reporting Template (CERT) [17] is presented in the Supplementary Material (Table S1). In our experience, the run-in period has contributed to improved adherence. The Consolidated Standards of Reporting Trials (CONSORT) diagram summarizing the process from screening to the end of the study is presented in the Supplementary Material (Figure S1). For the next 24 weeks, participants went to the fitness center for four sessions/week according to a schedule they determined. They exercised individually. Fitness center trainers introduced the exercise programs to the participants. All training sessions in both conditions consisted of 10–15 min of warm-up/cool down and 30–40 min of workout. Participants were contacted on a weekly basis by coaches to monitor their progress. To exercise at their target HR, participants wore a Polar Electro model s610i HR monitor during each training session. This monitor provided a digital display of HR and recorded HR throughout the training session. Data from each participant was downloaded into a computer located in each fitness center. Adherence to the training programs was documented by weekly logs and data from the HR monitors. A coach was assigned to each participant to monitor their performance and encourage compliance.

Aerobic Conditioning: Participants selected from a series of aerobic activities including treadmill, elliptical trainer, and stair stepper. For weeks 1 and 2 they trained at 55–65% of maximum HR as established during their qualifying CPET. In weeks 3 and 4, they increased their intensity to 65–75% of maximum HR and in weeks 5–24, they trained at 80% of maximum HR. 

Stretching/Toning: Under the guidance of their trainers and coaches, participants engaged in a series of stretches and toning exercises designed to promote flexibility and improve core strength. Core strengthening exercises included abdominal, back, and pelvic muscles. Participants were told to hold each stretch for 15–30 s (Table S2). Each stretch was repeated 10 times. Stretching exercises targeted a large range of muscle groups. In both cases, activities were not likely to result in improved aerobic capacity.

### 2.2. Study Outcomes

Cognitive function (the primary outcome) and aerobic capacity were assessed three times: prior to randomization into exercise/control conditions, and after 12 and 24 weeks. 

#### 2.2.1. Cardiorespiratory Exercise Test

Maximal oxygen uptake (VO_2_ max) was assessed by a graded exercise test (GXT) on an electronic-braked cycle ergometer (Lode Corival; Groningen, The Netherlands) which was connected to a metabolic measurement cart (Ultima CPXTM; MedGraphics, St. Paul, MN). Criteria for VO_2_ max were a respiratory exchange ratio >1.1, a plateau in the slope of the VO_2_-work-rate relationship (piece-wise linear regression analysis) [18], and an HR over the 90% of the maximum age-predicted HR limit. The equation HR_max_ = 220 – age was used to compute the maximum age-predicted HR limit [19]. The highest 15-breath moving median for the test was considered VO_2_ max [20].

#### 2.2.2. Cognitive Assessment

The Wechsler Test of Adult Reading (WTAR) [21] was administered at the initial assessment to estimate IQ. Tests were chosen from domains that are sensitive to aerobic exercise manipulations [2,3]. The specific measures used for each cognitive domain were selected prior to initiating analyses. This selection was validated using factor analysis of the baseline test data, which produced groupings comparable to those included cognitive domains listed below. Standardization of each test was based on mean and standard deviation of baseline values. Mean values of the standardized outcomes of the tests in each domain were used for analysis. Cogstate tasks [22] had multiple forms across visits; all remaining tasks remained the same. A full description of the selected tasks is contained in the original paper [6].

EF measures included a local switch costs from a Set switching task and total rule break errors across five trials of the Groton Maze Learning Test (CogState [22]). Episodic Memory measures included delayed recall from the Modified Rey Auditory Verbal Learning Test [23,24] and number of errors from the Continuous Paired Associate Learning task (CogState [22]). Processing Speed measures included number of symbols copied from the Digit Symbol task (WAIS-III Digit Symbol subtest [25]); number of clicks/second from the Groton Maze Chase Test (CogState [22]); and reaction time from the Identification Task (CogState [22]). Language measures included total words produced across three letters on the Controlled Oral Word Association [26] and animal names produced in 60 s on the Animal Naming [27]; attention was assessed with total number of correct cancellations within 5 min on the Two and Seven Test [28]. Working Memory was assessed with total number of correct sequence trials on the WAIS-III Letter Number Sequencing [29] and correct responses on the N-back task (CogState [22]). 

#### 2.2.3. Statistical Analysis

The primary analysis had been conducted to provide Class II evidence [30] of the effect of aerobic exercise on the cognitive domains. In each case the original models tested for moderation by age. The present analyses were designed to test for potential sex moderation in the effect of aerobic exercise on the cognitive domains. In the original report, additional analyses addressed the effect of aerobic exercise on aerobic capacity measured in VO_2_ max, and BMI. The present analyses focused on the additional sex moderation of these effects. We conducted the statistical analyses under the intention-to-treat analysis principles: all participants were analyzed as the being in condition they were assigned to at the randomization, regardless of their compliance to the exercise protocol. We conducted missing values pattern analysis, and we did not find specific patterns. Thus, we conducted mixed effects regression that allowing missing values in the outcomes under the assumption of missing completely at random.

Baseline demographic characteristics, VO_2_ max and cognitive variables were compared as a function of sex between the aerobic exercise and stretching/toning groups using the two-sample *t*-tests with unequal variances and Pearson’s chi-squared test for continuous and categorical variables, respectively.

Cognitive outcomes at baseline 12 and 24 weeks were compared between the groups using mixed effects models, adjusted for baseline age, years of education, and estimated IQ (which could be related to scores on cognitive tasks). This approach also adjusts for baseline performance when assessing change over time. Since our previous study reported an age moderation effect, we began with two-way and three-way interactions of group and time with baseline age. We then added group, time, sex and their two-way and three-way interactions as well as the four-way interaction between group, time, sex and baseline age. Baseline age was treated as a continuous variable after centering at age 40. This approach tested whether there were group differences in the change from baseline to week 12 or the change from baseline to week 24, and whether these differences are moderated by sex with and without controlling for age moderation. The parameters at weeks 12 and 24 were simultaneously estimated in the same model for each outcome. SAS software (version 9.4) was used for all statistical analyses. A two-sided *p* value of 0.05 or less was interpreted as a statistically significant result. When we found a significant effect of aerobic exercise on a cognitive outcome, we checked whether increments in VO_2_ max with exercise mediated the effect on EF. We conducted the mediation analysis using the mediation R-package [31] with 10,000 bootstrapping sampling. For the mediation analysis, we only included the final time point with age, sex, years of education, IQ, baseline VO_2_ max and EF adjusted.

#### 2.2.4. Data Availability

The data from this randomized clinical trial, including anonymized participant-level and study-level data (analyzable data sets) and other information (such as protocols), will be shared with qualified researchers as necessary for conducting legitimate research through direct request from the study principal investigators (Y.S. at ys11@columbia.edu or R.P.S. at rps7@cumc.columbia.edu).

## 3. Results

A total of 132 participants, age 20–67, were randomized equally into the stretching or aerobic conditions. Randomization was stratified by age and sex. Ninety-four of the 132 participants completed the full 6-month intervention. After randomization and the baseline assessment, 12 of the 132 participants did not begin the intervention. All people noted as dropped in the CONSORT diagram did not receive evaluations subsequent to dropping. The stretching condition contained 50 participants (34 women and 16 men) and the aerobic condition contained 44 participants (32 women and 12 men). Of these people, five participants (three in the AE and two in the stretching condition) completed all 96 sessions; 44% of the AE and 43% of the stretching condition completed more than 80% of the sessions. No adverse events occurred during exercise.

Descriptive statistics for the randomized participants at baseline are summarized in Table 1 by sex. Although there were more women than men in the study, the aerobic exercise and stretching/toning conditions were equally balanced for age and sex. Age, years of education, weight, height, and VO_2_ max at baseline significantly differed by sex.

Table 2 summarizes change from baseline to week 12 and 24 by exercise group and sex. Table 3 summarizes the F statistics for all of the three- and four-way interaction models used to explore the moderation effects reported below.

Compared to the stretching/toning group, the aerobic exercise group showed significantly improved VO_2_ max. There was no age or sex moderation (*p* > 0.9). Similarly, the aerobic exercise group showed reduced BMI, and there was no age (*p* = 0.08) or sex moderation (*p* = 0.80). 

**Figure 1:**
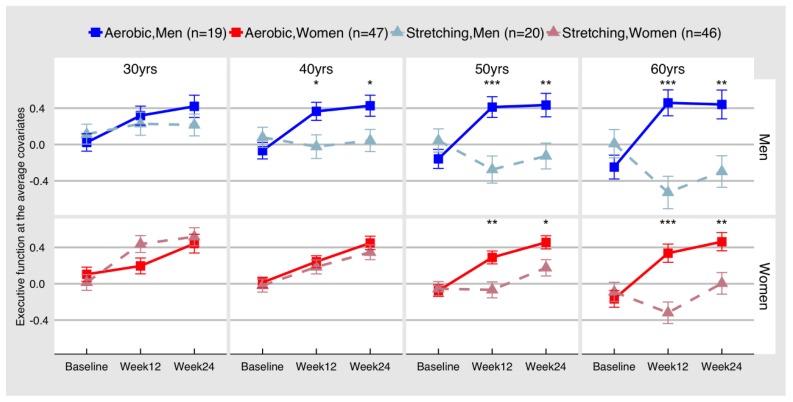
Change in executive function by condition, age and sex. Values are least squares means of fixed effects by age decades at the average IQ and years of education. There is significant age and sex moderation, such that older groups benefit from aerobic exercise more than young, and men benefit more than women. * *p* < 0.05; ** *p* < 0.01; *** *p* < 0.001

Change in EF by condition showed significant sex (F(2, 185) = 3.07, *p* = 0.0486) and age moderation (F(2, 183) = 10.84, *p* < 0.0001). Figure 1 demonstrates the combined effect of the age and sex moderation using least squares means of fixed effect by age decades at the average IQ and years of education. At each age, we compared group difference at each follow-up by age decade. In both groups EF improved more with greater age, and men benefited more than women at any age. More detailed parameter estimates are reported in Table 4a. 

**Figure 2:**
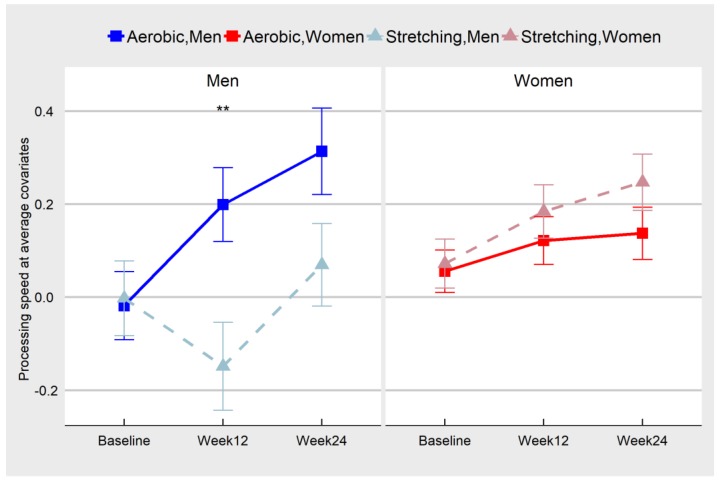
Change in processing speed by condition and sex. There was significant sex moderation while the age moderation was not significant. In men, processing speed in the aerobic group significantly improved by week 12 in comparison to the stretching group, but effect was diminished at week 24 due to slight improvement in the stretching group. In women, there was no aerobic exercise effect at either time point. * *p* < 0.05; ** *p* < 0.01; *** *p* < 0.001.

Processing speed showed significant sex moderation (F(2, 184) = 3.09, *p* = 0.0478) but not significant age moderation (F(2, 184) = 0.05, *p* = 0.9536). In men, processing speed in the aerobic group increased significantly by week 12 in comparison to the stretching/toning group (b = 0.35, *p* = 0.0051), but the effect was diminished at week 24 (b = 0.24, *p* = 0.0578) due to slight improvement in the performance of stretching/toning group. In women, there was no aerobic exercise effect at either time point (week 12: b = −0.06, *p* = 0.4156; week 24: b = −0.11, *p* = 0.1841). This effect is illustrated in Figure 2 and more detailed parameter estimates are reported in Table 4b.

No significant effects were noted for the other cognitive outcomes. In addition, for all cognitive outcomes, including EF and processing speed, there was no significant four-way interaction between age, sex, group and time (see Table 3, *p* > 0.05).

We tested whether the improvement in aerobic capacity mediated the improvement in EF and processing speed that were associated with the exercise intervention and whether this mediation differed by sex. Although the improvement in EF was mediated by improvement in aerobic capacity (indirect effect 0.0831, 95% CI: 0.002–0.20, *p* = 0.042), there was no significant sex moderation (indirect effect difference (men – women) 0.1183, 95% CI: −0.1644–0.4839, *p* = 0.45). For processing speed, there was no mediation by improvement in aerobic capacity (indirect effect 0.040, 95% CI: −0.0229–0.22, *p* = 0.22). Similarly, there was no differential mediation by sex (indirect effect difference (men – women) 0.0322, 95% CI: −0.1329–0.2344, *p* = 0.76).

## 4. Discussion

In this study, secondary analysis of a randomized controlled trial evaluating the efficacy of aerobic exercise for cognitive function in young and middle-age healthy adults, age 20 to 67, was conducted to explore potential sex moderation. In comparison to the stretching group, change in EF in the aerobic group showed significant sex and age moderation. In both men and women, EF improved more with greater age, and men benefited more than women at any age. Significant sex moderation was also observed for processing speed, as no change was observed in women, compared to a significant improvement by week 12 in men, which diminished in importance at week 24. We found no significant effects for the other evaluated cognitive domains (episodic memory, language, attention and working memory).

Two meta-analyses have reviewed sex moderation of cognitive effects in exercise trials. One found that “when the participant population was more than half female, the group as a whole showed greater benefit than if the population was at least half male” [3]. Using a similar approach of evaluating studies based on the preponderance of women in the sample versus the preponderance of men, another study also reported that three types of exercise interventions were all associated with greater cognitive performance of EF in women than men [14]. The present results suggest that the approach of evaluating differential sex-related effects based on the preponderance of men or women in a study could be misleading. Although our study participants were predominantly women, and there was an overall effect of aerobic exercise on EF, the exercise effect was significantly larger in men than in women. Thus, the appropriate meta-analytic approach would be to separate the effects by sex in included studies.

There have been several studies that have reported sex differences in the cognitive benefit from physical exercise or physical activity. A study of a large representative sample of elders found a relationship between reported light exercise and performance on five of six cognitive tests in men but not in women [7]. An epidemiologic study looking at five-year changes in cognition found that the effect of exercise differed by sex: women had a survival advantage compared with men, but men most benefited in cognitive functioning as measured by the Mini-Mental State Examination [9]. Based on a prospective study [10], lifelong intensive leisure-time physical activity was reported to reduce the rate of 12-year cognitive decline in the general population by around a third in men and a quarter in women. In another prospective study, independent of current physical activity, a significant association of early life physical activity with processing speed was only observed in men [11]. In older adults, the relation between cardiorespiratory fitness, and measure of resting state functional connectivity, such as the global efficiency (a measure of network integration), and the local efficiency (a measure of network specialization) of different brain networks appeared to be sex-dependent [12]. The positive association between local efficiency and cardiorespiratory fitness was more robust in men, but a negative association between global efficiency and cardiorespiratory fitness was also only measured in men. Two intervention studies found sex differences. One study [8] in young individuals (mean age 19.7 years) found that two 10-min sessions of moderate intensity exercise resulted in overall faster performance on a selective attention test in both men and women. Women showed better recognition memory than men, but there was improved object location memory only among men, and only among men who completed the memory task second. Another study did not note either an improvement in memory or sex moderation in improvement with a brief bout of moderate intensity exercise [32]. Lastly, an intervention study assessing the efficacy of a 6-month aerobic training in mitigating white matter hyperintensity progression in older adults with vascular cognitive impairment found that men who had completed the aerobic training intervention, in comparison to the women completing the same intervention, had significantly less white matter hyperintensity progression [13]. Thus, sex differences have been noted, but few intervention studies have specifically reported sex differences.

A review summarizes many of the factors that might influence sex differences in the response of memory to exercise intervention [33]. Several potential factors have been linked to sex moderation of physical exercise cognitive enhancement effects. One potential difference might relate to brain-derived neurotrophic factor (BDNF). A rodent study found that long-term voluntary wheel running resulted in increased total BDNF mRNA levels and BDNF protein in males, but not in females [34]. In a 12-year longitudinal study [35], the relationship in older adults between physical activity and cognitive performance was dependent in men but not in women on whether or not individuals were carriers of the methionine-specifying (Met) allele of the BDNF Val66Met polymorphism. The cognitive benefit associated with physical activity was observed in men *BDNF* met non-carriers, but not met carriers. No association was found in women regardless of their *BDNF* genotype. In older adults with subcortical ischemic vascular cognitive impairment, a 6-month of aerobic exercise program led to improvement in the EF set-shifting in women, but not in men. In the aerobic exercise group, women significantly increased their BDNF levels and a decrease in the BDNF levels was observed in men [36].

Other potential mechanisms underlying sex moderation of the exercise-cognition relationship include sex steroid hormones, sex-related difference in genetic and inflammatory risk factors, [37] glucose metabolism, cortisol level and Insulin-like Growth Factor-1 [38] and other physiological adaptations to exercise [39], such as cardiovascular adaptations [40]. 

All subjects in this study were cognitively intact, so the results have implications for healthy adults. At baseline, both EF and processing speed were poorer with age, as typically noted in cross-sectional studies of cognitive aging. In our original study, we noted age moderation for the effect of aerobic exercise on EF. Since baseline performance in this domain was poorer as a function of age, this finding suggests that aerobic exercise is more likely to improve age-related declines in EF rather than increase performance in those without a decline. Here we noted additional sex moderation, such that at any age men’s EF improved more than women’s EF. Here, we also noted an improvement in processing speed in men only. This improvement was not moderated by age, suggesting an actual increase in performance. Improvement in this domain was not noted in the original study, most likely because sex moderation was not considered. 

Reviews of exercise studies in older adults have noted improvements in multiple cognitive domains [5], several investigators have noted particular improvements in EF in adults over age 65 [41,42]. Memory performance is a common concern in the general population, and significant improvement in this domain was not noted. We did note some improvement in memory in men only, but it did not reach statistical significance. One review found that while physical activity enhances memory in rodents, findings are mixed in human studies [43]. Similarly, we did not find improvement in men or women in the remaining cognitive domains: language, attention and working memory. Better powered studies are needed to more definitively establish whether these cognitive functions might show improvement after consideration of sex moderation. Alternately, it would be important to establish that only specific cognitive functions improve with aerobic exercise. Still, both EF and processing speed are primary cognitive domains that are implicated in day-to-day performance and can decline with age, so our observations of improvement in these domains suggests that meaningful improvements in cognition can be achieved with aerobic exercise. 

There are several weaknesses to this study. Since this is a secondary analysis, the original study was not powered for comparing exercise effects in men and women. In addition, there were many more women participating in the study than men. Thus, we are reporting effects that are uncorrected for multiple comparisons; the reported interactions do not survive multiple comparison correction. It is also possible that power issues limit our ability to detect additional sex differences. For example, there is a suggestion in the data of an aerobic exercise effect for memory in men only, but the finding was at the borderline level. It is possible that well-powered studies might find sex moderation in other cognitive domains as well. In the primary report of this trial [6], we found that cortical thickness increased significantly in the aerobic exercise group in a left frontal region and did not interact with age. However, that analysis used only a subset of the full data, and we were not adequately powered to assess sex moderation. We used the maximum HR to prescribe exercise intensity. While some exercise studies have used this measure, it would have been preferable to use the heart rate reserve (HRR), which considers the resting HR and this allows better individualization. Although study adherence was quite good, the potential effects of exercise on cognition might be stronger with better adherence. Finally, this study included adults, age 20 to 67. The finding suggests that potential sex moderation should be evaluated across the entire age range. 

The present study highlights the importance of using sex-disaggregated analyses when assessing the cognitive benefit of physical exercise interventions. Larger, more appropriately powered studies are called for to better understand the biological and social mechanisms (gender roles, relations and inequalities) by which these sex-related cognitive benefit differences may arise in the context of physical exercise intervention. Future studies should include equal distributions of men and women, and be adequately powered to detect sex differences in the benefit of aerobic exercise. The present findings suggest that men benefited more than women, but we feel that this should be replicated in larger scale studies. It will be also be important for future studies to assess whether sex differences may be more influential at one period of life than another [44]. In addition, the future studies should directly test some of the underlying mechanisms that may account for sex differences. This should include both BDNF concentrations as well as *BDNF* genotype. Similarly, a series of inflammatory risk factors should be evaluated. Imaging studies could help to determine whether there are sex differences in the change in brain volume or cortical thickness that has previously been noted in exercise studies. Similarly, sex differences in change in the integrity of the default mode network should be evaluated, since these changes have also been associated with aerobic exercise. The proposed approach is particularly compatible with the development of precision medicine [45] where exercise recommendations could be individualized to achieve maximal cognitive benefit.

## Figures and Tables

**Table 1:** Baseline characteristics of the participants.

	Aerobic Condition		Stretching/Toning Condition		Sex Difference ^1^
	Women (*n* = 47)	Men (*n* = 19)	Women (*n* = 46)	Men (*n* = 20)	
Age, y	43 (12.9)	38.1 (10.2)	41.9 (15.0)	33.5 (11.3)	0.0039 **
Education, y	16.2 (2.6)	14.8 (2.5)	16.6 (1.9)	15.8 (2.7)	0.0306 *
Estimated IQ	111.8 (12.8)	113.9 (12.9)	111 (13.8)	112.1 (17.1)	0.566
Weight, lb	156.7 (36.3)	194.2 (31)	152.7 (36.5)	187 (26.3)	<0.0001 ***
Height, in	64 (3.2)	69.6 (2.2)	64.3 (3.1)	69.3 (3.9)	<0.0001 ***
Body mass index	26.6 (5.3)	28.1 (4.1)	25.9 (5)	27.4 (3.9)	0.072
VO_2_ max, (mL/kg/min)	25.44 (6.3)	33.06 (7.48)	26.6 (5.87)	33.77 (5.63)	<0.0001 ***
Processing Speed	–0.02 (0.79)	−0.14 (0.83)	0.11 (0.71)	0.11 (0.83)	0.7210
Episodic memory	0.14 (0.73)	−0.29 (0.92)	0.10 (0.80)	−0.01 (1.04)	0.1404
Working memory	–0.01 (0.78)	0.18 (0.63)	0 (0.75)	0.05 (0.75)	0.3671
Language	0.06 (0.95)	−0.12 (0.58)	0.09 (0.93)	0.05 (0.71)	0.4365
Attention	0.14 (0.63)	−0.15 (1.18)	0.03 (0.77)	−0.27 (1.35)	0.1750
Executive function	−0.02 (0.74)	−0.18 (0.94)	−0.04 (0.54)	0.36 (0.67)	0.3952
TIADL total error	0.62 (0.68)	0.74 (1.24)	0.28 (0.54)	0.50 (0.76)	0.3551
Race *n* (%)					
White	19 (40.43)	11 (57.89)	16 (34.78)	12 (60)	0.0886 ^2^
Black	16 (34.04)	5 (26.32)	9 (19.57)	3 (15)	
Others	12 (25.53)	3 (15.79)	21 (45.65)	5 (25)	

Abbreviations: TIADL = Timed instrumental activities of daily living tasks; cognitive and functional measures are presented as z scores; values are mean (SD); higher values are always better; ^1^ two sample *t*-test with unequal variance was used to compare sex difference; ^2^ chi-square test was used; * *p* < 0.05; ** *p* < 0.01; *** *p* < 0.001.

**Table 2:** Change from baseline to week 12 and 24 by exercise group and sex.

		Aerobic				Stretching			
		Women		Men		Women		Men	
	Week	***n***		***n***		***n***		***n***	
VO_2_ Max (mL/kg/min)	12	38	3.3 (4.78)	15	4.48 (6.02)	33	0.33 (3.54)	14	0.82 (5.56)
	24	30	3.35 (4.96)	11	3.25 (4.42)	28	0.25 (4.41)	14	0.84 (6.22)
Executive Function	12	38	0.29 (0.58)	16	0.42 (0.52)	38	0.19 (0.85)	14	0.02 (0.37)
	24	32	0.5 (0.59)	12	0.52 (0.3)	34	0.35 (0.74)	16	0.05 (0.73)
Attention	12	38	−0.45 (1.15)	16	−0.08 (1.05)	38	−0.19 (0.65)	13	−0.36 (1.23)
	24	32	−0.65 (2.23)	12	−0.41 (1.44)	34	−0.51 (1.19)	16	−0.06 (0.93)
Language	12	38	0.09 (0.59)	16	0.08 (0.51)	38	0.01 (0.51)	14	0.15 (0.49)
	24	32	0.13 (0.58)	12	0.12 (0.57)	34	0.2 (0.69)	16	0.23 (0.54)
Processing Speed	12	38	0.06 (0.38)	16	0.21 (0.46)	38	0.12 (0.52)	14	−0.14 (0.51)
	24	32	0.06 (0.47)	12	0.33 (0.42)	34	0.19 (0.43)	16	0.06 (0.51)
Episodic Memory	12	38	0.27 (0.59)	16	0.36 (0.51)	38	0.32 (0.52)	14	0.1 (0.87)
	24	32	0.36 (0.59)	12	0.59 (0.52)	34	0.58 (0.54)	16	0.26 (0.96)
Working Memory	12	38	0.15 (0.88)	16	0.06 (0.46)	38	0.38 (1.09)	14	0.2 (0.73)
	24	32	0.43 (0.67)	12	0.22 (0.53)	34	0.36 (0.6)	16	0.35 (0.85)
BMI	12	36	−0.5 (1.4)	16	−0.51 (1.19)	36	−0.12 (0.97)	14	−0.17 (1.54)
	24	31	−0.85 (1.2)	12	−1.05 (1.77)	31	−0.07 (0.97)	15	−0.67 (1.49)

Values are mean (SD).

**Table 3:** F-statistics for all three-way and four-way interaction models.

	Three-Way Interaction Models				Four-Way Interaction Model	
	Age × Session × Group		Sex × Session × Group		Age × Sex × Session × Group	
	**F**	***p*-Value**	**F**	***p*-Value**	**F**	***p*-Value**
VO_2_ max	0.06	0.9426	0.09	0.9097	1.59	0.2065
Executive Function	10.84	<0.0001 **	3.07	0.0486 *	0.77	0.4639
Attention	0.47	0.6242	0.68	0.5099	1.03	0.3595
Episodic Memory	0.81	0.4445	2.22	0.1112	1.34	0.2656
Language	0.43	0.6531	0.12	0.8833	0.42	0.6554
Speed	0.05	0.9536	3.09	0.0478 *	0.83	0.4368
Working Memory	0.07	0.9305	0.66	0.5173	0.48	0.6203
BMI	2.55	0.0808	0.23	0.7987	0.42	0.6571

* *p* < 0.05; ** *p* < 0.001.

**Table 4:** Least square means differences for performance of aerobic exercise vs. stretching toning groups on executive function (Table 4a) and processing speed (Table 4b), as illustrated in Figure 1 and Figure 2. Numbers of subjects at each visit are listed in Table 2.

**Table 4a Aerobic—Stretching Executive Function**							
**Age**	**Session**	**Sex**	**Estimate**	**S.E**	***t***	***p***	
30	Baseline	W	0.09	0.12	0.77	0.4445	
		M	−0.09	0.14	−0.63	0.5278	
	12 Weeks	W	−0.24	0.13	−1.90	0.0585	
		M	0.09	0.16	0.55	0.5839	
	24 Weeks	W	−0.08	0.14	−0.53	0.5987	
		M	0.21	0.17	1.20	0.2335	
40	Baseline	W	0.03	0.09	0.37	0.7122	
		M	−0.15	0.14	−1.02	0.3097	
	12 Weeks	W	0.06	0.10	0.57	0.5674	
		M	0.39	0.16	2.36	0.0192	*
	24 Weeks	W	0.10	0.11	0.93	0.3531	
		M	0.38	0.17	2.25	0.0253	*
50	Baseline	W	−0.02	0.10	−0.21	0.8325	
		M	−0.20	0.17	−1.22	0.2255	
	12 Weeks	W	0.36	0.11	3.18	0.0017	**
		M	0.69	0.19	3.63	0.0004	***
	24 Weeks	W	0.28	0.12	2.44	0.0158	*
		M	0.56	0.19	2.89	0.0043	**
60	Baseline	W	−0.08	0.14	−0.56	0.5786	
		M	−0.26	0.20	−1.26	0.2076	
	12 Weeks	W	0.66	0.15	4.28	<0.0001	***
		M	0.99	0.23	4.29	<0.0001	***
	24 Weeks	W	0.46	0.15	2.97	0.0034	**
		M	0.74	0.24	3.13	0.0021	**
**Table 4b Aerobic—Stretching Processing Speed**							
**Session**	**Sex**	***n*** **^1^**	**Estimate**	**S.E.**	***t***	***p***	
Baseline	M	39	−0.02	0.11	−0.15	0.8836	
	W	93	−0.02	0.01	−0.24	0.8120	
12 Weeks	M	30	0.35	0.12	2.83	0.0051	**
	W	76	−0.06	0.08	–0.82	0.4156	
24 Weeks	M	28	0.244	0.13	1.91	0.0578	
	W	66	−0.11	0.08	−1.33	0.1841	

Significance of least square means differences for performance of aerobic exercise vs. stretching toning groups: * *p* < 0.05; ** *p* < 0.01; *** *p* < 0.001; ^1^ The sample size at each time point over the two groups. Further detailed breakdown by group is in Table 2.

## Supplementary Materials

The following are available online at https://www.mdpi.com/2077-0383/8/6/886/s1, Table S1: List of stretching exercises, Table S2: CERT checklist, Figure S1: CONSORT diagram
