# Supplementary material for: Sex Moderates the Effect of Aerobic Exercise on Some Aspects of Cognition in Cognitively Intact Younger and Middle-Age Adults
Source: J Clin Med. 2019 Jun 21;8(6):886. doi: 10.3390/jcm8060886 (PMC6617072; doi:10.3390/jcm8060886)
Supplement: Supplementary file 1 [file jcm-08-00886-s001.zip › supplementary/Table S1 List of stretching exercises.pdf]

List of stretching exercises

- Abdominal Stretch
- Back Stretches
- Butterfly Stretch
- Chest Stretch
- Chest Stretch against Wall
- Cross-Legged Stretch
- Double Calf Stretch on Step
- Figure 4 Stretch
- Fire Log Stretch
- Hamstring Stretch
- Happy Baby
- High Lunge Stretch
- Iliotibial (IT) Band Stretch
- Kneeling Back Stretches
- Kneeling Chest Stretch
- Kneeling Full Body Stretch
- Kneeling Lateral Stretch
- Kneeling Twist
- Knees to Chest
- Low Lunge Stretch
- Lying Double Leg Twist
- Lying Twist
- Modified Abdominal Stretch
- Neck Stretch with Rotation
- Neck Stretches
- Overhead Triceps Stretch
- Pyramid Stretch
- Quad Stretch
- Reaching Side Bend
- Reverse Hurdler's Stretch
- Runner's Lunge
- Seated Glute Stretch with Twist
- Seated Glute Stretch with Twist Variation
- Seated Hamstring Stretch
- Shoulder Stretch
- Side Squat
- Single Calf Stretch on Step
- Single Knee to Chest
- Squat Stretch
- Squat Stretch Variation
- Standing Full Body Stretch
- Standing Hamstring and Calf Stretch
- Standing Hamstring Stretch
- Standing Hamstring with IT Stretch
- Standing IT Stretch with Overhead Reach
- Standing Quad Stretch
- Upper Body Stretch
- Wall Calf Stretch
- Wall Calf Stretch Variation
- Wrist Stretches
