# Supplementary material for: Sex Moderates the Effect of Aerobic Exercise on Some Aspects of Cognition in Cognitively Intact Younger and Middle-Age Adults
Source: J Clin Med. 2019 Jun 21;8(6):886. doi: 10.3390/jcm8060886 (PMC6617072; doi:10.3390/jcm8060886)
Supplement: Supplementary file 1 [file jcm-08-00886-s001.zip › supplementary/Table S2 CERT checklist.pdf]

CERT ✓ Consensus on Exercise Reporting Template

A Checklist for what to include when reporting exercise programs

Section/Topic	Item #	Checklist item	Location **	
			Primary paper (page, table, appendix)	† Other (paper or protocol, website (URL)
**WHAT: materials**	1	Detailed description of the type of exercise equipment (e.g. weights, exercise equipment such as machines, treadmill, bicycle ergometer etc)	p.3, l.105	
**WHO: provider**	2	Detailed description of the qualifications, teaching/supervising expertise, and/or training undertaken by the exercise instructor	p.3, l.104	
**HOW: delivery**	3	Describe whether exercises are performed individually or in a group	p.3, l.97	
	4	Describe whether exercises are supervised or unsupervised and how they are delivered	p.3, l.104	
	5	Detailed description of how adherence to exercise is measured and reported	p.3, l.103	
	6	Detailed description of motivation strategies	p.3, l.105	
	7a	Detailed description of the decision rule(s) for determining exercise progression	p.3, l.107	
	7b	Detailed description of how the exercise program was progressed	p.3, l.107	
	8	Detailed description of each exercise to enable replication (e.g. photographs, illustrations , video etc)	Supplementary	
	9	Detailed description of any home program component (e.g. other exercises, stretching etc)	N/A	
	10	Describe whether there are any non-exercise components (e.g. education, cognitive behavioural therapy, massage etc)	N/A	
	11	Describe the type and number of adverse events that occurred during exercise	p.5, l.191	

**WHERE: location**	12	Describe the setting in which the exercises are performed	p.2, l.89
**WHEN, HOW MUCH: dosage**	13	Detailed description of the exercise intervention including, but not limited to, number of exercise repetitions/sets/sessions, session duration, intervention/program duration etc	p.2, l.96
**TAILORING: what, how**	14a	Describe whether the exercises are generic (one size fits all) or tailored whether tailored to the individual	p.3, l.107
	14b	Detailed description of how exercises are tailored to the individual	p.3, l.107
	15	Describe the decision rule for determining the starting level at which people commence an exercise program (such as beginner, intermediate, advanced etc)	p.3, l.107
**HOW WELL: planned, actual**	16a	Describe how adherence or fidelity to the exercise intervention is assessed/measured	p.3, l.103
	16b	Describe the extent to which the intervention was delivered as planned	p.2, l.94

***It is recommended that this checklist is used in conjunction with the Explanation and Elaboration Statement which is a guide each item in the CERT Checklist**

The CERT Checklist is designed for reporting details of an exercise intervention. The CERT Checklist should be used in conjunction with a reporting checklist appropriate for the study type e.g. the CONSORT Statement (www.consort-statement.org) for randomised controlled trials, the SPIRIT Statement (www.spirit-statement.org) for a clinical trial protocol. For further guidance regarding reporting guidelines please consult the EQUATOR network (www.equator-network.org)

** Authors – please use N/A if an item is not applicable Reviewers – please use ”?” if information is not provided or not/insufficiently reported

† If the information is not provided in the primary paper that is under consideration, please provide details of where this information is available e.g. in a published protocol, published papers (provide citation details) or on a website (provide the URL).
