# Supplementary figures and images for: Sex Moderates the Effect of Aerobic Exercise on Some Aspects of Cognition in Cognitively Intact Younger and Middle-Age Adults
Source: J Clin Med. 2019 Jun 21;8(6):886. doi: 10.3390/jcm8060886 (PMC6617072; doi:10.3390/jcm8060886)

# CONSORT Diagram

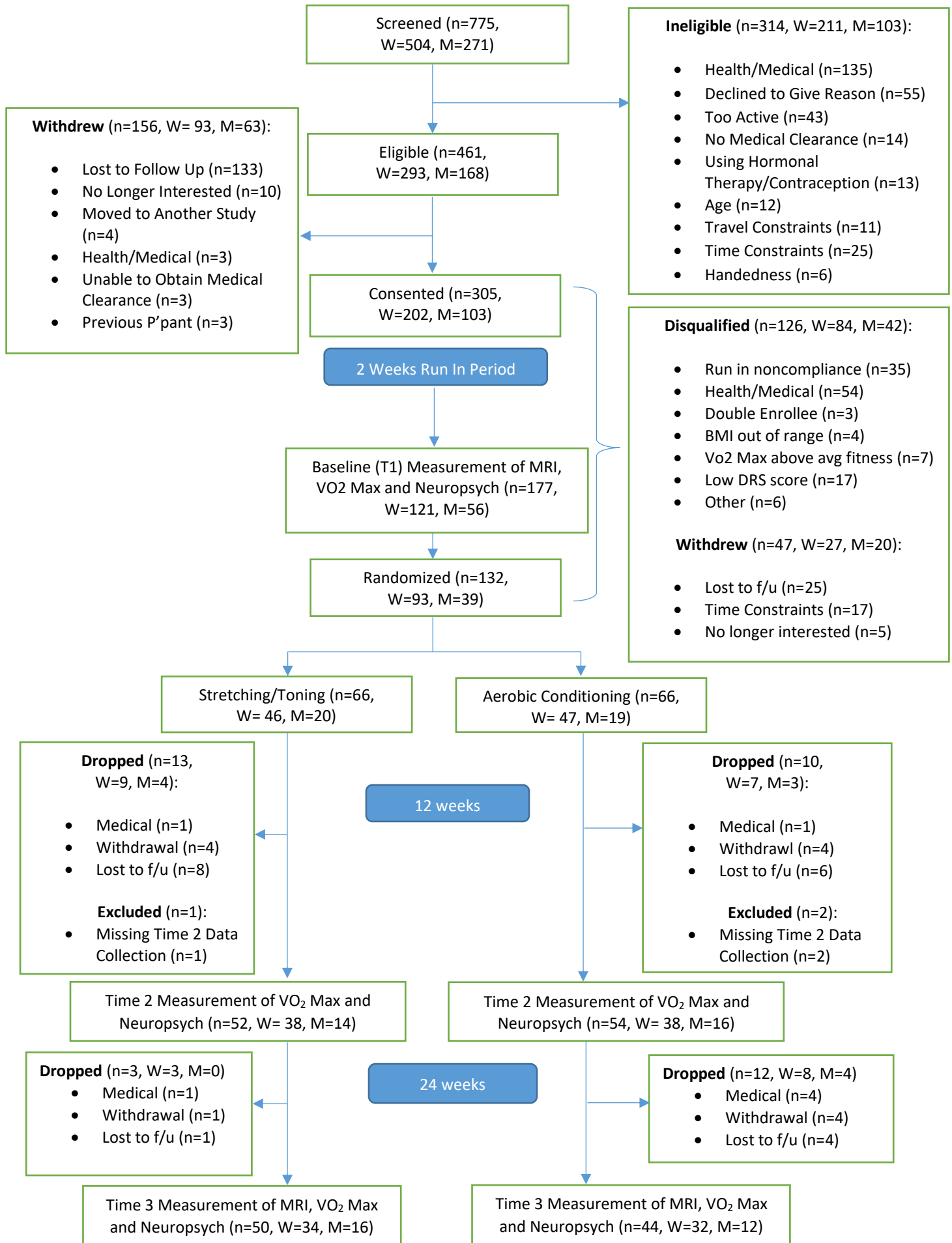

Supplement: Supplementary file 1 [file jcm-08-00886-s001.zip › supplementary/Figure S1 CONSORT.pdf]
